# Role of liraglutide in Alzheimer’s disease pathology

**DOI:** 10.1186/s13195-021-00853-0

**Published:** 2021-06-12

**Authors:** Maria Vargas-Soria, Maria Jose Carranza-Naval, Angel del Marco, Monica Garcia-Alloza

**Affiliations:** 1 Division of Physiology, School of Medicine, Instituto de Investigacion Biomedica de Cadiz (INIBICA), Universidad de Cadiz, Edificio Andres Segovia, C/Dr. Maranon 3, 3er piso, Cadiz, Spain; 2 Salus Infirmorum-Universidad de Cadiz, Cadiz, Spain

**Keywords:** Liraglutide, Alzheimer’s disease, Amyloid, Tau, Inflammation, Cognition

## Abstract

**Background:**

The described relationship between Alzheimer’s disease (AD) and type 2 diabetes (T2D) and the fact that AD has no succesful treatment has led to the study of antidiabetic drugs that may limit or slow down AD pathology.

**Main body:**

Although T2D treatment has evident limitations, options are increasing including glucagon-like peptide 1 analogs. Among these, liraglutide (LRGT) is commonly used by T2D patients to improve β cell function and suppress glucagon to restore normoglycaemia. Interestingly, LRGT also counterbalances altered brain metabolism and has anti-inflammatory properties. Previous studies have reported its capacity to reduce AD pathology, including amyloid production and deposition, tau hyperphosphorylation, or neuronal and synaptic loss in animal models of AD, accompanied by cognitive improvement. Given the beneficial effects of LRGT at central level, studies in patients have been carried out, showing modest beneficial effects. At present, the ELAD trial (Evaluating Liraglutide in Alzheimer’s Disease NCT01843075) is an ongoing phase IIb study in patients with mild AD. In this minireview, we resume the outcomes of LRGT treatment in preclinical models of AD as well as the available results in patients up to date.

**Conclusion:**

The effects of LRGT on animal models show significant benefits in AD pathology and cognitive impairment. While studies in patients are limited, ongoing clinical trials will probably provide more definitive conclusions on the role of LRGT in AD patients.

## Background

Alzheimer’s disease (AD) is the most common cause of dementia. Type 2 diabetes (T2D) may increase the risk to suffer AD over two-fold [1, 2]. However, it remains unclear whether T2D and AD are parallel phenomena or synergistic diseases linked by vicious pathological cycles [3]. In this sense, findings in patients relating T2D to AD classical pathology are inconsistent [4]. However, T2D increases the risk to develop AD, even after adjusting for vascular risk factors [5, 6] and if only 10% of diabetic patients end up suffering AD, the number of AD patients will double [2]. This situation and the fact that AD has no successful treatment supports the study of antidiabetic drugs that may reduce or slow down AD pathology.

Liraglutide (LRGT) is a glucagon-like peptide 1(GLP-1) analog that has been widely assessed in animal models of AD. Initial studies with patients have shown modest beneficial effects and it is currently under evaluation in the ELAD trial (Evaluating Liraglutide in Alzheimer’s Disease NCT01843075). We have reviewed available bibliography on the potential mechanisms through which LRGT may benefit AD. An overall improvement of brain metabolic alterations, amyloid (Aβ) and tau pathologies, inflammation, and neuronal damage are observed in animal models, supporting further studies in patients.

## Main text

### Preclinical studies

Glucagon-like peptide 1 (GLP-1) is implicated in the control of glycemia and metabolic homeostasis, both in the periphery and the central nervous system [7, 8]. Due to the importance of GLP-1 signaling on cognitive function [9] and the relationship between AD and T2D [10], GLP-1 analogs, and LRGT specifically, may provide a relevant venue to ameliorate AD pathology [11]. Previous studies have shown some controversial outcomes in AD models. Whereas brain weight [12, 13], hippocampal insulin [13–16], cortical glucose levels or brain GLUT1 and GLUT4 [13] do not seem to be affected, LRGT treatment increases GLP1-receptors in the hippocampus of AD mice [15–17]. Similarly, LRGT also increases insulin receptor levels in a primate model of AD [14], although no differences have been observed in AD mice [18]. LRGT ameliorates insulin resistance in the hippocampus by reducing phosphorylated insulin receptor levels [19, 20] and insulin receptor substrate-1 [19, 20]. Interestingly, insulin degrading enzyme, that is reduced in AD preclinical models and a feasible underlying mechanism for AD and T2D [21, 22], is preserved or increased in the cortex and hippocampus from AD mice after LRGT treatment [18, 20].

**Fig. 1:**
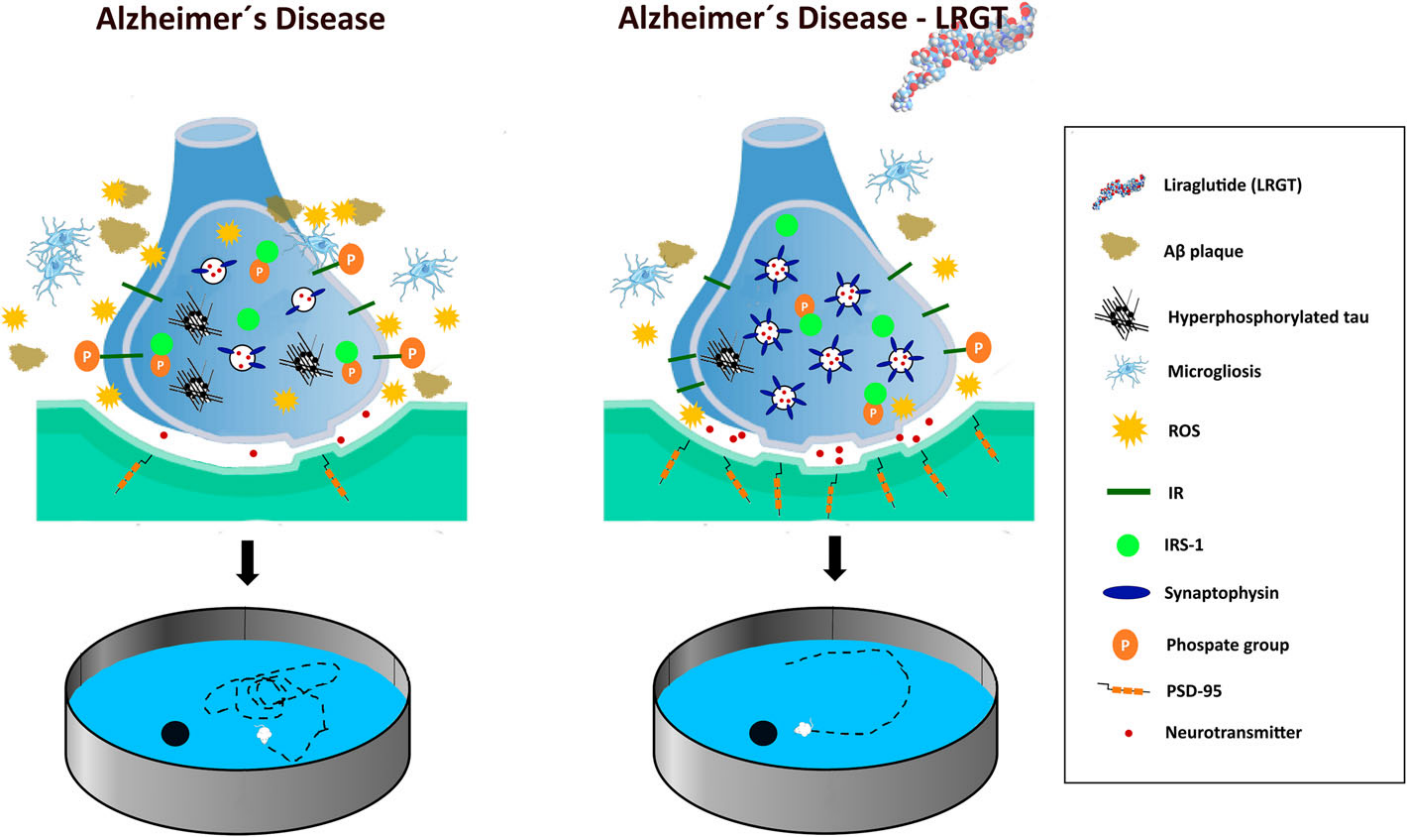
Schematic effects of liraglutide in the brain with AD pathology

Autopsy cohort studies have revealed a limited role of T2D on classical AD neuropathological features (amyloid (Aβ) plaques and tau tangles) [4]. However, studies in animals show an overall improvement after different administration protocols [12, 13, 23, 24], including prophylactic [23] and long-term treatments [23, 25–27]. Whereas some studies have reported no effects on amyloid pathology [28], the majority of the results show that LRGT dramatically reduces Aβ plaque size [29], number [19, 29], and burden [18, 23, 30–32]. LRGT also decreases Aβ aggregates [30] and restores increased levels of β-secretase 1 and presenilin 1 [17] in the brain from an AD mice, once Aβ pathology is fully established (Fig. 1). Moreover, positive effects have also been observed when LRGT is administered before Aβ plaques deposit [23]. In addition, LRGT limits tau hyperphosphorylation by modulating the activity of ERK and JNK in 3xTgAD [12, 13], APP/PS1 [29], and hTauP301L mice [33]. Likewise, LRGT reduces hippocampal tau phosphorylation by modulating Akt and GSK-3β [15], and in hyperhomocysteinemic rats, tau hyperphosphorylation is reduced through the activation of PP2Ac [17]. Likewise, LRGT neuroprotection is mediated by a reduction of neurofilament phosphorylation in 3xTgAD animals [12]. LRGT also improves synaptic plasticity [18, 34], density [14], structure [15, 27], and synapsis number [35], increasing synaptophysin and PSD-95 levels in AD mice [23, 30, 35] together with increased long-term potentiation and paired-pulse facilitation [18, 30, 34, 35]. NMDA synapse-associated proteins are restored by LRGT in the hippocampus from hyperhomocysteinemic rats [17], and cAMP/PKA pathway is also improved [14, 34]. Additionally, LRGT not only attenuates neural loss and degeneration, but it also increases neurogenesis in the cortex [18, 23, 26, 35] and the subventricular zone [31, 32], reduces the number of degenerating cells in the cortex and hippocampus [12], and increases cell proliferation in the dentate gyrus of AD animals [26]. Inflammation is also a major feature in AD and previous studies show that LRGT reduces microgliosis and astrocytosis in the cortex [19, 23, 30, 35] and hippocampus [29, 31, 32]. Besides, LRGT limits pro-inflammatory cytokines, including TNF-α, IL-1ß, or IL-10 [13, 24]. In line with these observations, LRGT also decreases brain oxidative stress, by reducing glucose-6-phosphate dehydrogenase activity, the formation of cortical carbonyl groups, nitrite and 8-hydroxy-2′-deoxyguanosine in 3xTgAD mice [13]. Similarly, oxidative phosphorylation of cortical astrocytes is reduced in 5xFAD mice [27] (Fig. 1).

The positive effects of LRGT on AD-like pathology support the beneficial role of LRGT on learning and memory in most of the studies. In this sense, spatial working memory improves after LRGT treatment [12, 15, 17, 18, 24], and LRGT also restores episodic memory in AD models [14, 18, 23] (Fig. 1). In line with these observations, contextual fear conditioning [14], active-avoidance T-maze task [25], or clasping behavior [33] are also improved by LRGT, while locomotor activity does not seem affected [12, 17, 23].

### Studies in AD patients

The above described outcomes in preclinical models of AD have set the basis to futher assess LRGT in patients. Whereas other antidiabetic drugs, including GLP-1 analogs or dipeptidyl peptidase 4 inhibitors, have been part of preceding or ongoing clinical trials, studies with LRGT specifically are still limited. Previous meta-analysis has shown a pro-cognitive class effect of antidiabetic agents in AD/mild cognitive impairment, although the actual beneficial effects with LRGT are limited [36]. LRGT administration to individuals with subjective cognitive complaints, at risk for AD, improves intrinsic connectivity within brain areas. While this did not translate into cognitive differences between study groups after 12 weeks of treatment [37], other studies have shown that treatment with LRGT to AD patients for 6 months raises blood-brain glucose transfer capacity, restoring glucose transport [38], as an initial requirement to improve brain alterations. Gejl et al. [39] (ClinicalTrials.govNCT01469351) have also reported that treatment with LRGT to AD patients for 6 months prevents cerebral metabolic rate of glucose consumption decline, as an indicator of cognitive impairment, synaptic dysfunction, and disease evolution. Whereas Aβ load or cognition do not seem to be affected, the authors state the study was underpowered. Another study with pre- or early diabetes patients has recently shown that LRGT improves short-term memory and memory composite in treated patients [40]. The ELAD trial is presently ongoing and the main objectives include evaluation of glucose metabolic consumption in cortical regions and cognition, MRI changes, microglial activation, and amyloid or tau changes [41], and the latest results will be published shortly.

## Conclusions

Preclinical studies show beneficial effects of LRGT on AD pathological features and cognition. While the studies in patients have only shown moderate positive effects, the ongoing ELAD trial may provide relevant insights on the actual role of LRGT at central level and open new venues of treatment for AD patients.

## Data Availability

Not applicable.

## Abbreviations

Aβ: Amyloid-β
AD: Alzheimer’s disease
T2D: Type 2 diabetes
GLP-1: Glucagon-like peptide 1
LRGT: Liraglutide
